# Efficacy and safety of umbilical cord mesenchymal stem cells in treatment of cesarean section skin scars: a randomized clinical trial

**DOI:** 10.1186/s13287-020-01695-7

**Published:** 2020-06-25

**Authors:** Dazhi Fan, Meng Zeng, Qing Xia, Shuzhen Wu, Shaoxin Ye, Jiaming Rao, Dongxin Lin, Huishan Zhang, Huiting Ma, Zhongchao Han, Xiaoling Guo, Zhengping Liu

**Affiliations:** 1 Foshan Institute of Fetal Medicine, Affiliated Foshan Maternity & Child Healthcare Hospital, Southern Medical University, 11 Renminxi Road, Foshan, 528000 Guangdong China; 2 Department of Obstetrics, Affiliated Foshan Maternity & Child Healthcare Hospital, Southern Medical University, 11 Renminxi Road, Foshan, 528000 Guangdong China; 3 Department of Epidemiology and Biostatistics, School of Public Health, Anhui Medical University, Hefei, 230032 Anhui China; 4 Menzies Institute for Medical Research, University of Tasmania, Private Bag 23, Hobart, Tasmania 7000 Australia; 5 State Key Laboratory of Experimental Hematology, Institute of Hematology & Blood Diseases Hospital, Chinese Academy of Medical Sciences & Peking Union Medical College, Tianjin, 300020 China

**Keywords:** Umbilical cord mesenchymal stem cells, Cesarean section, Skin scars, Randomized controlled trial

## Abstract

**Background:**

Pathological skin scars, caused by cesarean section, affected younger mothers esthetically and psychosocially and to some extent frustrated obstetricians and dermatologists. Umbilical cord mesenchymal stem cells (UC-MSCs), as a population of multipotent cells, are abundant in human tissues, providing several possibilities for their effects on skin scar tissues. Herein, we performed a randomized, double-blind, placebo-controlled, three-arm clinical trial, aiming to assess the efficacy and safety of UC-MSCs in the treatment of cesarean section skin scars among primiparous singleton pregnant women.

**Methods:**

Ninety primiparous singleton pregnant women undergoing elective cesarean section were randomly allocated to receive placebo, low-dose (3 × 10^6^ cells), or high-dose (6 × 10^6^ cells) transdermal hydrogel UC-MSCs on the surface of the skin incision. The primary outcome was cesarean section skin scars followed after the sixth month, assessed by the Vancouver Scar Scale (VSS).

**Results:**

All the participants completed their trial of the primary outcome according to the protocol. The mean score of estimated total VSS was 5.52 in all participants at the sixth-month follow-up, with 6.43 in the placebo group, 5.18 in the low-dose group, and 4.71 in the high-dose group, respectively. No significant difference was found between-group in the mean scores for VSS at the sixth month. Additional prespecified secondary outcomes were not found with significant differences among groups either. No obvious side effects or adverse effects were reported in any of the three arms.

**Conclusion:**

This randomized clinical trial showed that UC-MSCs did not demonstrate the effects of improvement of cesarean section skin scars.

**Trial registration:**

ClinicalTrials.gov identifier, NCT02772289. Registered on 13 May 2016.

## Background

Cesarean delivery has been increasing worldwide over recent years, especially in China [1, 2]. It has become the most common surgical intervention in many countries for pregnant women [3]. Due to its association with adverse outcomes for mothers and babies [4], cesarean delivery has become a great concern globally. Meanwhile, pathological skin scars after cesarean section were a big problem esthetically and psychosocially among younger mothers, and to some extent, frustrating obstetricians and dermatologists.

Pathological skin scars, such as hypertrophic scar, keloids, and atrophic, are aberrant fibro-metabolic diseases resulting from severe burn injury, blunt force trauma, wound infection, or other surgical interventions [5]. They are characterized by the proliferation of fibroblasts, accumulation of collagen, and infiltration of inflammatory cells [6, 7]. Epidemiology studies indicated that the pathological skin scars were found in 33 to 91% in subjects who suffered from burn injury and 39 to 68% in cases who received surgery [8]. Moreover, numerous sufferers have different degrees of psychosocial disturbance, varying from mild to severe [9]. Currently, therapeutic strategies, such as occlusive dressings, compression therapy, surgical excision, topical or intralesional corticosteroids, radiotherapy, lasers, cryotherapy, and a combination with other adjuvant topical drugs, are commonly used for alleviating the severity of skin scars [5, 7, 10, 11]. However, most of the aforementioned therapies are ineffective or with unsatisfactory effects, including high recurrence rates, hypo- or hyper-pigmentation, telangiectasia, blisters, dermal atrophy, inflammation, erosion, superficial ulceration, crusting, erythema, edema, burning sensation, and pain at injection site [7].

Stem cells, especially mesenchymal stem cells (MSCs), representing a population of multipotent cells, are abundant in human tissues. They have aroused immense interests for clinical applications because of their easy availability and potential for recovering [12, 13]. Substantial animal and clinical studies have shown that MSCs can inhibit pathological fibrosis in many organs, such as the heart, lung, and kidney, and therefore can improve the prognosis of many diseases, such as liver injury, spinal cord injury, acute respiratory distress syndrome, blood disease, and critical limb ischemia [14–19]. These provided a possibility of skin scar tissue repair affected by MSCs.

MSCs, owing to their multifunctional roles, can migrate to the wound sites directionally. They formed a part of microenvironment, improved wound healing, and inhibited pathological skin scars [20]. Among them, UC-MSCs are most easily accessible, with low immunogenicity, and can be expanded in vitro [21]. UC-MSCs can be easily isolated and collected from the umbilical cord by using an accessible procedure [22]. Previous researches have shown that MSCs can suppress proliferation and activation of keloid fibroblasts and inhibit extracellular matrix synthesis through a paracrine signaling mechanism [23, 24], and thus may be a novel topical agent for pathological skin scar treatment. Liu and his colleges [16] found that transplantation of MSCs via the ear artery can inhibit the hypertrophic scarring in a rabbit ear hypertrophic scar model significantly, indicating that MSCs may have potential clinical applications in modulating the process of wound healing. Similarly, another research in a rabbit model has demonstrated that local injection of MSCs efficiently prevented hypertrophic scar formation by regulating inflammation [15]. In a case report, researchers found that hydrogel with MSCs can accelerate wound healing and ameliorate the condition of the wound in a diabetic patient within only 3 weeks [25]. Taken together, these studies suggested that MSCs can regulate the process of wound healing and prevent pathological skin scar formation.

In our present work, we conducted a randomized, double-blind, placebo-controlled clinical trial in order to assess the efficacy and safety of umbilical cord mesenchymal stem cells (UC-MSCs) in the treatment of cesarean section skin scars among primiparous singleton pregnant women. The effectiveness of UC-MSCs on treating cesarean section skin scars, to our knowledge, has not been reported yet in the literature. Our hypothesis was that UC-MSCs can improve the degree of scar repairing and increase the satisfaction of patients.

## Methods

Primiparous singleton pregnant women undergoing elective cesarean section in the Affiliated Foshan Maternity & Child Healthcare Hospital, Southern Medical University, a tertiary university-affiliated hospital, were screened for participation in this double-blind, three-arm, randomized, controlled trial. The number of deliveries in our hospital has stabilized at approximately 12,000 per year [26]. This trial has been registered at the ClinicalTrials.gov (registration number NCT02772289) and approved by the Ethics Committee of the Affiliated Foshan Maternity & Child Healthcare Hospital, Southern Medical University.

The trial protocol was designed by the trial executive committee and further approved by the institutional review board of the hospital. The trial protocol has been published in an international peer-reviewed journal in the field of clinical trials [27] and presented at an international conference on stem cell and regenerative medicine [28]. The trial UC-MSCs (1 × 10^6^ cells each pump) and matching placebo were donated from the Health-Biotech Pharmaceutical Company (Beijing, China). The company was not involved in the trial design, data analysis, manuscript preparation, publication, or presentation of the trial.

The UC-MSCs were isolated from the umbilical cord of a healthy donor after informed consent by the method described above [22, 25]. Briefly, the umbilical cord tissue was washed with phosphate-buffered saline (PBS) and then minced into 1–2 mm^3^ fragments. These tissue pieces were then incubated with 0.075% collagenase (Sigma, St Louis, MO, USA) for 30 min and then 0.125% trypsin (Gibco, Grand Island, NY, USA) for 30 min with gentle agitation at 37 °C. Fetal bovine serum (FBS) (Hyclone, Logan, UT, USA) was added to neutralize the excess trypsin. The digested mixture was then passed through a 100-μm filter to obtain cell suspensions. The cells were plated at a density of 1 × 10^6^ cells/cm^2^ in non-coated T-25 or T-75 cell culture flasks. Growth medium consisted of Dulbecco’s modified Eagle’s medium with low glucose (DMEM-LG; Gibco) and 5% fetal bovine serum, supplemented with 10 ng/mL vascular endothelial growth factor (VEGF; Sigma), 10 ng/mL epidermal growth factor (EGF; Sigma), 100 U penicillin/streptomycin (Sigma), and 2 mM l-glutamine (Gibco). Cell cultures were maintained in a humidified atmosphere with 5% CO_2_ at 37 °C. After 3 days of culture, the medium was replaced and non-adherent cells were removed. The medium was then changed twice weekly thereafter until 60–80% confluence had been reached. The obtained adherent cells were replated at a density of 1 × 10^4^/cm^2^.

The UC-MSCs released from the cell banking were cultured and expanded in the good manufacturing practice (GMP) laboratory to prepare final cell products. They were checked to be sterile and free from human immunodeficiency virus, cytomegalovirus, syphilis, mycoplasma, hepatitis B virus, hepatitis C virus, Epstein–Barr virus, and endotoxins. The CD73, CD105, CD90, CD151, CD166, Oct4, nestin, and Sox2 are highly expressed (> 95%), and CD45, CD34, CD31, CD11b, CD184, and HLA-DR are negative. The final UC-MSCs were activated and mixed with sodium alginate powder, resulting in a gelatinous UC-MSC complex. The UC-MSCs can be stored for a year in a − 80 °C refrigerator, and the post-transplantation cell survival should be more than 80%.

From November 2016 to September 2018, pregnant women were referred to the study team for screening. The eligibility of enrollment was confirmed by investigators. Primiparous singleton pregnant women aged 21 to 35 years between the 37th and 42nd weeks of gestation were eligible for enrollment if they were going to have a programmed cesarean delivery. Pregnant women with a gel allergy were excluded because of the difficulty to complete the trial in women with the symptom. Women who were planning to have any other cosmetic procedure to the skin incision during the study period and who had a history of smoking, wounds or local disease in the treatment area, recent or current cancer history, any systemic uncontrolled disease, or presented with a keloid formation were excluded. Women unwilling to adhere to the trial protocol were also excluded. The supplementary appendix provided the trial inclusion and exclusion criteria. Written informed consent was obtained from all the included subjects.

Routine blood examinations for aspartate aminotransferase (AST), alanine aminotransferase (ALT), uric acid (UA), blood urea nitrogen (BUN), and tumor markers (CA125, CA153, CA199, and CEA) were carried before skin incision. Meanwhile, physical examinations, past medical histories, and pigmentation and erythema around the skin incision were subsequently measured and recorded.

Eligible participants who signed informed consent forms were then randomly assigned in a 1:1:1 ratio, by means of a computer algorithm, to allocate placebo, low-dose (3 × 10^6^), or high-dose (6 × 10^6^) groups. Transdermal hydrogel was occupied immediately after skin incision suturing. The participants received a total of six hydrogels on the surface of the skin incision, once a day. Participants in the placebo group received placebo hydrogel for 6 days; those in the low-dose group will receive one dose of hydrogel with cells for three consecutive days and then placebo hydrogel for the next three consecutive days; those in the high-dose group will receive cells for 6 days.

The primary outcome of cesarean section skin scars was assessed at 6 months after enrollment by using Vancouver Scar Scale (VSS). Four parameters were used in the evaluation of the severity of cesarean section skin scars: pigmentation (normal, hypopigmented, mixed, or hyperpigmented), vascularity (normal, pink, red, or purple), pliability (normal, supple, yielding, firm, ropes, or contracture), and height (flat, < 2 mm, 2–5 mm, or > 5 mm) [29]. Each parameter contained ranked subscales that may be summed to obtain a total score ranging from 0 to 14, with 0 representing normal skin. The higher the total score, the more the severity of the scarring. The outcome at the first and third months after enrollment was also assessed.

Additional prespecified secondary outcomes included adverse effects, wound healing at 14 days, erythema and pigmentation around the skin incision, skin scar area and volume, immunoglobulins (IgG, IgA, IgM) and complement component (C3, C4) in milk, and satisfaction of treatment (an overall assessment of the treatment, as measured on a 5-point scale [with “very good,” “good,” “moderate,” “slight,” or “none”] assessed by the questionnaire of “would you recommend this method of treatment to a friend?”).

### Statistical analysis

The sample size was calculated according to the primary outcome of VSS. The score of VSS was evaluated according to professor Yu-Chen Huang’s preliminary trial in Taipei Medical University WanFang Hospital [30]. The sample size of this anticipated trial was 74, and the final sample size was increased to 90 (*n* = 30 in each group) to achieve 90% power to detect 1.5 difference of the VSS in the MSC group and the placebo group. Data analysis was performed using R 3.1 software.

All the results were analyzed according to the intention-to-treat principle. Standard descriptive methods were used to summarize the trial participants overall and by intervention group. Continuous variables were demonstrated by mean ± standard deviation and calculated by one-way analysis of variance (ANOVA). Categorical variables were demonstrated by a relative number and calculated by chi-square test. The primary outcome was assessed among all women who had the follow-up on the sixth month according to the intention-to-treat principle. After homogeneity test of characteristics of the participants at baseline among groups, the difference (with 95% confidence interval) of VSS of women between the MSC group and placebo group was calculated using two-sided *t* test and *F* test. After correcting of multiple comparisons by Bonferroni correction, the differences were considered significant at *α* = 0.017.

Subgroup analysis of the primary outcome according to participants’ demographics and clinical characteristics was prespecified. The analyses were stratified according to gestational age and maternal age. Two-sided *t* test, *F* test, chi-square test, or Fisher’s exact test was used to compare the groups in all secondary analyses; the results were presented without adjustment for multiplicity and should be considered exploratory. All the data was analyzed by SPSS statistical software.

## Results

**Fig. 1:**
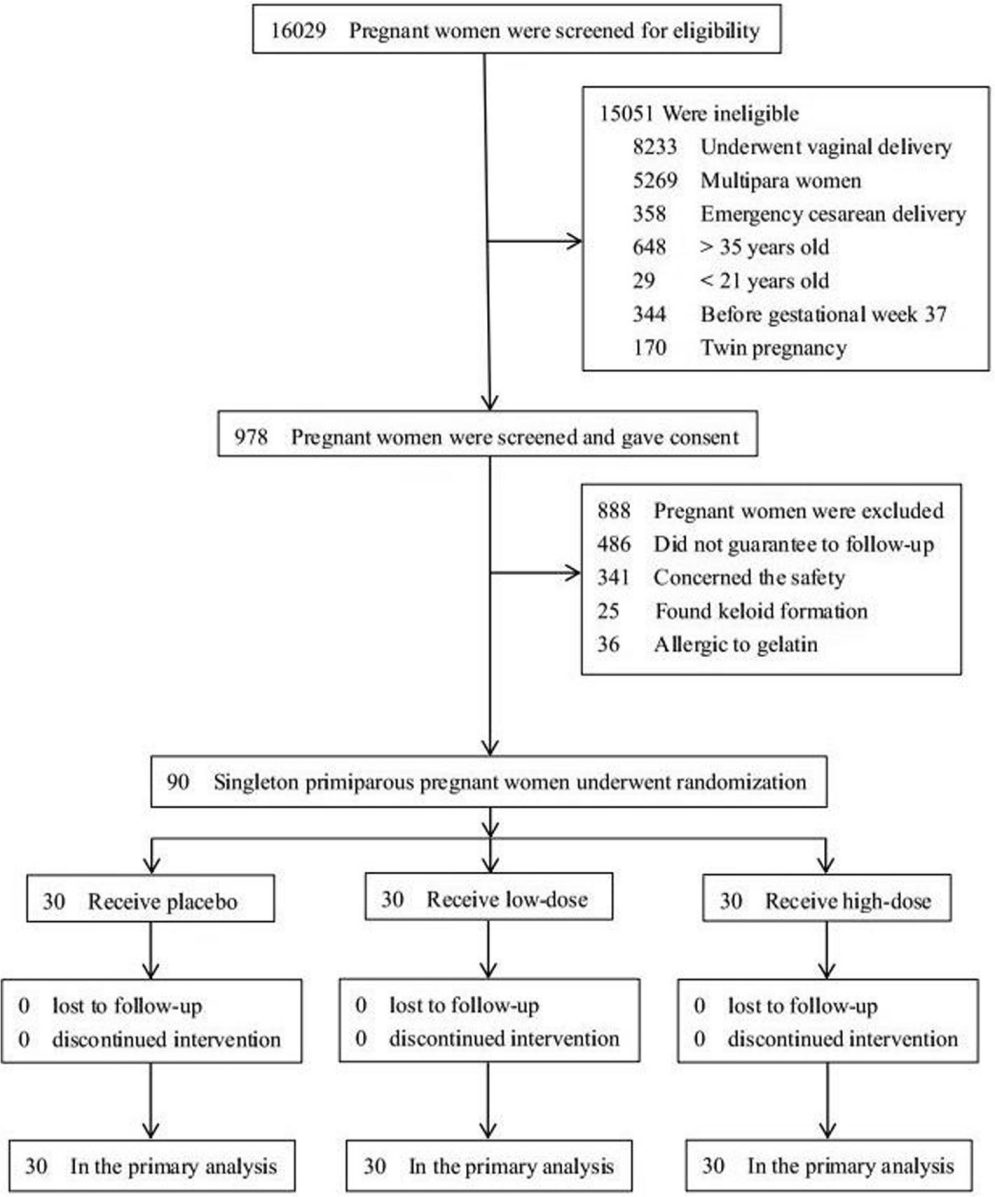
Enrollment and outcomes

From November 2016 to September 2018, a total of 16,029 pregnant women gave birth in the department and 7796 (48.64%) women underwent cesarean delivery. In these cesarean births, there were 5269 multipara women, 358 emergency cesarean sections, 677 too old (> 35 years) or too young (< 21 years), 344 preterm delivery (before gestational week 37), and 170 twin pregnancy. Of the 978 potential eligible participants, 486 pregnant women planned to go to other cities after delivery, 25 pregnant women were found with keloid formation, 36 women were allergic to gelatin, and 341 women declined to participate because of safety concerns. Finally, 90 eligible singleton primiparous pregnant women were enrolled and underwent randomization, with 30 women in each group (Fig. 1). Participant recruitment closed when the sample size reached ninety.

All the participants were occupied to the trial, and the follow-ups of the primary outcome were finished according to the protocol. The mean age and gestation were 28.04 years and 39.47 weeks, respectively. The mean time of operation and the length of incision were 39.20 min and 14.53 cm, respectively. The mean value of pigmentation and erythema was 165.37 and 265.43, respectively. No abnormal liver and kidney indexes were found in all participants. Women randomized to each of the three groups were well balanced regarding demographic and delivery characteristics (Table 1).

**Table 1:** Characteristics of the participants at baseline [mean ± SD or *n* (%)]

	All participants	Placebo group	Low-dose group	High-dose group	*F*/*χ*^2^	*p* value
Age (years)	28.04 ± 3.39	28.01 ± 3.82	28.34 ± 3.05	27.79 ± 3.34	0.197	0.821
Age group						
21–27	45 (50.0)	17 (56.7)	12 (40.0)	16 (53.3)	1.867	0.500
28–35	45 (50.0)	13 (43.3)	18 (60.0)	14 (46.7)		
Gestation (week)	39.47 ± 1.10	39.50 ± 1.07	39.30 ± 1.25	39.61 ± 0.97	0.603	0.550
Gestation group						
37–39 week	59 (65.6)	20 (66.7)	20 (66.7)	19 (63.3)	0.098	0.999
40–42 week	31 (34.4)	10 (33.3)	10 (33.3)	11 (36.7)		
Operation time (min)	39.20 ± 10.45	39.17 ± 12.48	38.48 ± 7.87	39.93 ± 10.81	0.137	0.873
Incision length (cm)	14.53 ± 1.07	14.36 ± 1.23	14.73 ± 0.93	14.52 ± 1.05	0.881	0.418
Pigmentation	165.37 ± 73.15	167.99 ± 69.86	160.07 ± 81.14	168.13 ± 70.00	0.116	0.890
Erythema	265.43 ± 77.03	264.43 ± 77.75	271.99 ± 84.63	259.69 ± 69.93	0.188	0.829

**Fig. 2:**
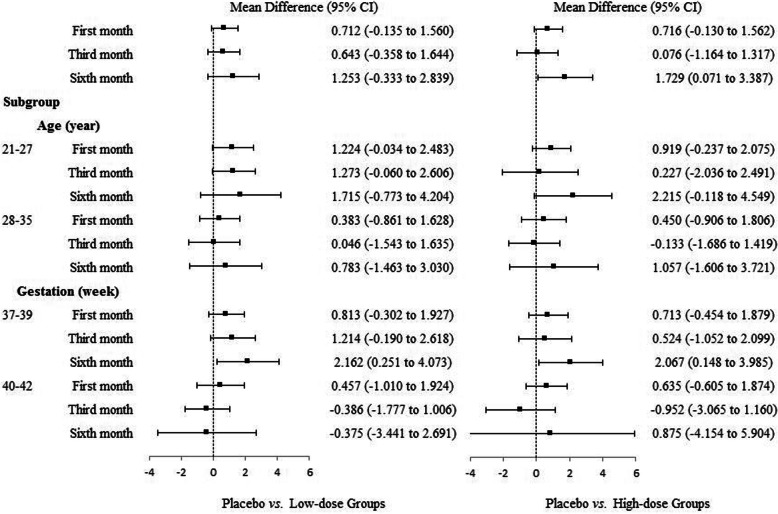
Mean difference of the Vancouver Scar Scale between the MSC and placebo group

**Fig. 3:**
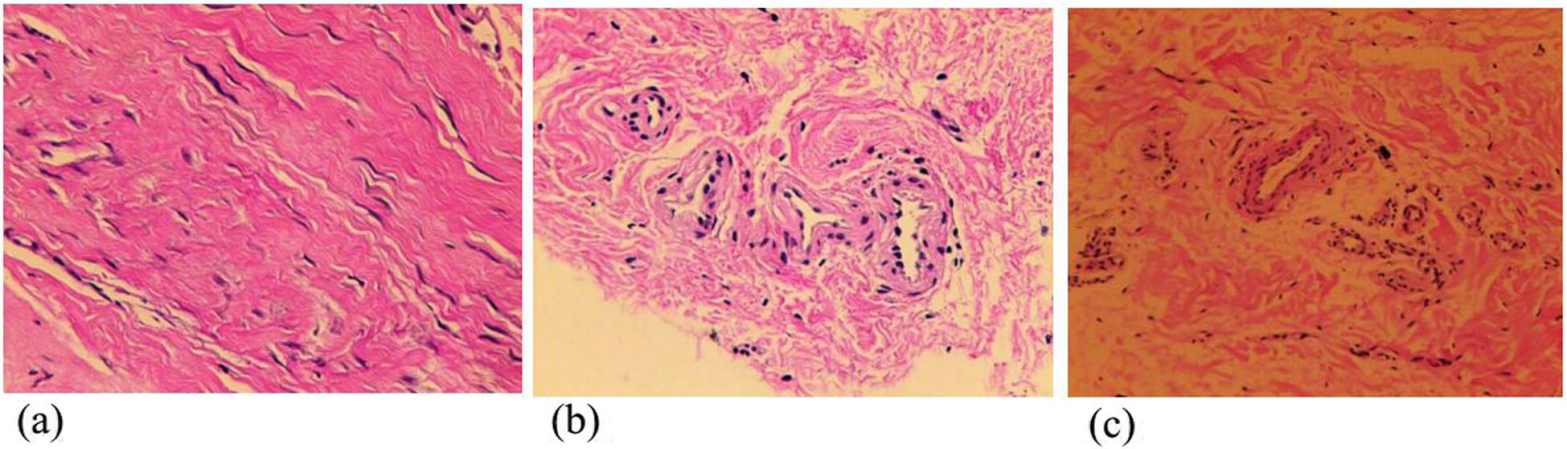
Representative hematoxylin-eosin (H&E) staining of cesarean section skin scars with the three groups. **a** Placebo group. **b** Low-dose group. **c** High-dose group

The mean total VSS score was 5.52 in all participants at the sixth month, with 6.43 in the placebo group, 5.18 in the low-dose group, and 4.71 in the high-dose group, respectively. No significant difference was found between-group in the mean scores of VSS at the sixth month. Similar results were also shown among groups at the first and third months (Table 2 and Fig. 2). Representative hematoxylin-eosin (H&E) staining pictures are shown in Fig. 3.

**Table 2:** Vancouver Scar Scale and satisfaction outcomes among participants

	All participants	Placebo group	Low-dose group	High-dose group	*F*/*χ*^2^	*p* value
VSS						
First month	4.68 ± 1.49	5.17 ± 1.40	4.46 ± 1.53	4.46 ± 1.47	1.852	0.164
Third month	5.20 ± 1.74	5.48 ± 1.69	4.83 ± 1.63	5.40 ± 1.96	0.899	0.413
Sixth month	5.52 ± 2.70	6.43 ± 2.48	5.18 ± 2.79	4.71 ± 2.66	2.362	0.103
Satisfaction						
Very good	0 (0.0)	0 (0.0)	0 (0.0)	0 (0.0)	3.279	0.214
Good	0 (0.0)	0 (0.0)	0 (0.0)	0 (0.0)		
Moderate	0 (0.0)	0 (0.0)	0 (0.0)	0 (0.0)		
Slight	42 (46.7)	14 (46.7)	11 (36.7)	21 (70.0)		
None	48 (53.3)	16 (53.3)	19 (63.3)	9 (30.0)		

*VSS* Vancouver Scar Scale

For all additional prespecified secondary outcomes, there were no significant differences among the three groups. Detailed research data and statistical results were provided in the attachment files. No obvious side effects or adverse effects, such as fever, chills, pain, inflammation, and wound infection, were reported in any of the three arms. Moreover, renal and liver functions and tumor markers, including CA125, CA153, CA199, and CEA, were all within the normal range (Table 3).

**Table 3:** The value of the liver, kidney, and tumor markers before and at the sixth month [mean ± SD or median (Q25–Q75)]

	Baseline				Sixth month			
	All participants	Placebo group	Low-dose group	High-dose group	All participants	Placebo group	Low-dose group	High-dose group
ALT	9.72 ± 3.60	10.17 ± 4.04	9.63 ± 3.12	9.37 ± 3.65	16.82 ± 12.70	17.05 ± 9.97	17.09 ± 17.67	16.13 ± 6.54
AST	15.48 ± 3.40	15.93 ± 3.18	15.30 ± 3.04	15.20 ± 3.96	17.37 ± 6.34	16.62 ± 5.08	17.78 ± 7.94	17.75 ± 5.48
UA	345.58 ± 89.29	342.90 ± 83.96	328.40 ± 88.22	365.43 ± 94.42	333.10 ± 70.49	322.52 ± 62.01	343.48 ± 80.41	332.06 ± 67.91
BUN	3.77 ± 2.63	4.03 ± 4.42	3.61 ± 0.82	3.66 ± 0.93	4.65 ± 1.15	4.61 ± 1.01	4.74 ± 0.99	4.57 ± 1.55
CA125	19.63 (15.21–26.75)	19.62 (15.19–25.54)	19.36 (14.65–30.49)	20.75 (15.36–31.66)	15.13 (9.87–19.72)	15.85 (9.65–20.39)	13.18 (9.07–18.53)	15.13 (9.45–22.67)
CA153	13.21 (10.56–16.07)	13.20 (10.70–16.87)	12.81 (10.78–16.32)	13.39 (9.68–15.76)	8.62 (6.55–12.30)	7.52 (6.27–11.86)	8.89 (6.41–12.44)	10.49 (7.33–12.44)
CA199	9.54 (6.91–14.48)	9.29 (6.38–15.42)	8.66 (7.03–19.38)	10.27 (7.03–14.47)	9.11 (6.48–14.45)	10.58 (6.13–15.45)	7.53 (6.53–15.76)	10.78 (6.42–14.13)
CEA	0.62 (0.31–0.87)	0.70 (0.30–0.85)	0.60 (0.38–0.90)	0.61 (0.21–0.83)	0.84 (0.53–1.48)	1.21 (0.54–1.59)	0.66 (0.51–1.39)	0.79 (0.60–1.38)

*ALT* alanine aminotransferase, *AST* aspartate aminotransferase, *BUN* blood urea nitrogen, *CA125* cancer antigen 125, *CA153* carbohydrate antigen 153, *CA199* carbohydrate antigen 199, *CEA* carcinoembryonic antigen, *UA* uric acid

By the end of the trial period, 51.00% (46) of the women described their experience overall as “slight” and 49.00% (44) described as “none.” None of them described as “moderate,” “good,” or “very good.” However, the majority of women (90% (81)) stated that they would use the intervention again if there are any changes and would recommend this treatment method to their friends (Table 2).

## Discussion

Based on the findings of the present work, UC-MSCs did not demonstrate the effects of improvement of cesarean section skin scars. No significant difference was found between groups in terms of the VSS score, secondary and adverse-effect outcomes at the first-, third-, and sixth-month follow-ups.

In a case-control prospective study by Abo-Elkheir et al., the authors proved that MSCs can effectively enhance wound regeneration in deep burns, representing a new modality providing great hope for burn injury patients [31]. A randomized phase 1/2 clinical trial focusing on skin expansion showed that it was safe and effective to intradermally transplant autologous stem cells to promote mechanical stretch-induced skin regeneration in vivo and was a feasible clinical application to provide amounts of tissue for complex skin defect [32]. Unfortunately, we could not observe significant cesarean section skin scar improvement based on VSS rating and other outcomes, such as wound healing at the second week, pigmentation and erythema around the incision, and skin area and volume. We considered that the insufficient effective dose may be a key important cause. Most of the previous effective clinical studies used the doses of 10^8^ or 10^9^ cells [33, 34].

The number of cells, 10^6^, seems inadequate in this trial. What was more regrettable was that there were a number of cells, about 10%, which performed inactively. The reason that we focused on 10^6^ UC-MSCs was the full consideration of the safety for those sensitive population, namely pregnant women. Although there was no statistical difference, the present study found that the VSS rating was lower with the dose increased. Understanding this appearance is of great significance for future clinical trials that target higher doses of UC-MSCs to intervene cesarean section skin scar.

The biological function of UC-MSC hydrogel was complex, and the greater potential may be related to paracrine, immunomodulation, and the vascular differentiation capacity of these cells. Several studies have shown that MSCs can secrete multiple cytokines and growth factors, including stromal cell-derived factor 1, basic fibroblast growth factor (bFGF), TGF-β, IGF-1, VEGF, and HGF, and increase angiogenesis and collagen accumulation in the wound tissue [35–37]. It has been reported that MSC hydrogel can stimulate wound tissue to generate granulation tissues [25, 38]. Similarly, in this study, the histologic evaluation showed that the capillary density of skin scar was increased in UC-MSC groups. In addition, in vivo evidence revealed that MSCs markedly expressed the anti-inflammatory factor IL-10 and suppressed the production of IL-6, IL-8, IL-1, IL-4, TNF-α, interferon-γ, and ICAM-1 [35, 39, 40]. This indicated that MSCs can induce high immunomodulation activity and reduce inflammation during wound healing.

Another reason for negative findings may be due to insufficient follow-up period. Some studies have suggested that it usually took 18–24 months for a typical scar to mature [11]. Considering a low rate of lost to follow-up in the special group of pregnant women, we only followed for 6 months at this design. Satisfactorily, all the participants completed the trial, with a primary outcome according to the protocol at the sixth month. In the next step, we will further follow up to observe the long-term efficacy in these participants.

Women with keloid formation may benefit from this therapy. However, in order to control the confounding deviation as much as possible, we excluded these women in the trial. These women should be the focus of the next study. In addition, our used stem cells were derived from allogeneic cells supplied by the company manufacturing. If possible, trials using autologous stem cells may yield better effectiveness.

A major concern with MSC therapy is the safety potential. In the present study, many eligible pregnant women who declined to participate in the trial are concerned about the short- and long-term safety of MSCs, especially the concern regarding their future breastfeeding. To date, most of the published preclinical and clinical trials have reported that MSCs are safe when being used for the treatment of injuries to the central nervous system, biliary lesions, musculoskeletal disease, digestive system disease, and cardiovascular disease [17, 18, 33, 34, 41].

Meanwhile, a meta-analysis that included 36 articles, involving a total of 1012 participants, evaluated the safety of MSC-based therapy in all prospective clinical trials [42]. This study did not detect any risk like malignancy, infection, organ system complications, and acute infusion toxicity in treated participants. On the other hand, this study proved that intravascular infusions of MSC therapy appeared to be safe. Our findings were consistent with the evidence from the above studies that the use of MSCs in pregnant women is safe.

Interestingly, although participants in both the placebo and MSC groups were both not satisfied with the skin scar at the sixth month, most of them still want to try it again. It means that these younger mothers have gradually accepted MSC therapy after this clinical trial and looked forward to it.

## Conclusions

In conclusion, in this three-arm RCT trial involving women with primiparous singleton pregnancies, the UC-MSC-treated group was not significantly different from the placebo group for the reduction of cesarean section skin scar and neither did the increasing recognition of participants’ satisfaction.

## Supplementary information

**Additional file 1.**

## Data Availability

The datasets used and/or analyzed during the current study are available from the corresponding author on reasonable request.

## Abbreviations

MSCs: Mesenchymal stem cells
RCT: Randomized clinical trial
VSS: Vancouver Scar Scale
UC-MSCs: Umbilical cord mesenchymal stem cells
