# Supplementary material for: Efficacy and safety of umbilical cord mesenchymal stem cells in treatment of cesarean section skin scars: a randomized clinical trial
Source: Stem Cell Res Ther. 2020 Jun 25;11:244. doi: 10.1186/s13287-020-01695-7 (PMC7316165; doi:10.1186/s13287-020-01695-7)
Supplement: Supplementary file 1 — Additional file 1. [file 13287_2020_1695_MOESM1_ESM.pdf]

Supplementary Appendix

Table S1. The details of the trial inclusion and exclusion criteria…………………………….. 2
Table S2. Schedule of visits and assessments………………………………………………. 2
Table S3. REEDA outcome among participants………………………………………………… 3
Table S4. Pigmentation outcome among participants……………………………………………. 4
Table S5. Erythema outcome among participants……………………………………………….... 5
Table S6. Scar area (mm^2) outcome among participants……………………………………….. 6
Table S7. Scar volume (mm^3) outcome among participants……………………………………. 7
Table S8. Immunoglobulins (IgG, IgA, IgM) and complements (C3, C4) in milk outcomes among participants………………………………………………………………………… 8

Figure S1. Mean difference of REEDA between MSCs and placebo group………………….. 3
Figure S2. Mean difference of pigmentation between MSCs and placebo group……………… 4
Figure S3. Mean difference of Erythema between MSCs and placebo group…………………. 5
Figure S4. Mean difference of Scar Area (mm^2) between MSCs and placebo group………… 6
Figure S5. Mean difference of Scar volume (mm^3) between MSCs and placebo group……… 7
Figure S6. Mean difference of immunoglobulins (IgG, IgA, IgM) and complements (C3, C4) in milk between MSCs and placebo group…………………………………………. 9

Table S1. The details of the trial inclusion and exclusion criteria

Inclusion criteria	Exclusion criteria
Primiparous women receiving elective cesarean delivery	Any systemic uncontrolled disease
Ages between 21-35 years	Recent or current cancer
Gestation ages ≥ 37 weeks and < 42 weeks	History or presenting with a keloid formation
Willing to give and sign an informed consent form and a photographic release form	Wounds or local disease in treatment area
Willing to comply with study dosing and complete the entire course of the study	Planning any other cosmetic procedure to the study area during the study period
	Smoking
	Gel allergy

Table S2. Schedule of visits and assessments

Time point (weeks)	2	4	12	24
VSS		√	√	√
REEDA	√			
Erythema		√	√	√
Pigmentation		√	√	√
Scar area		√	√	√
Scar volume		√	√	√
Immunoglobulin		√	√	√
Satisfaction				√
Adverse events	√	√	√	√

VSS: Vancouver Scar Scale;

REEDA: Redness, Edema, Ecchymosis, Discharge, Approximation.

Table S3. REEDA outcome among participants.

	All participants	Placebo group	Low-dose group	High-dose group	F/χ^2	*P*
	1.13±0.66	1.26±0.76	1.19±0.62	0.97±0.57	1.540	0.221
Subgroup						
Age (year)						
21-27	1.10±0.74	1.33±0.90	1.00±0.45	0.93±0.70	1.259	0.295
28-35	1.17±0.58	1.17±0.58	1.31±0.70	1.00±0.39	1.085	0.348
Gestation (week)						
37-39	1.07±0.58	1.17±0.51	1.12±0.70	0.95±0.52	0.728	0.488
40-42	1.24±0.79	1.44±1.13	1.30±0.48	1.00±0.67	0.787	0.466

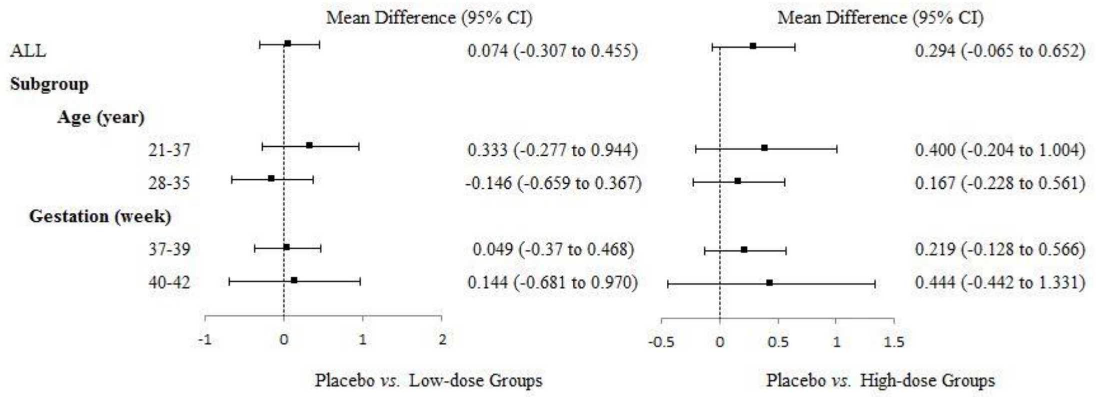

Figure S1. Mean difference of REEDA between MSCs and placebo group.

Table S4. Pigmentation outcome among participants.

	All participants	Placebo group	Low-dose group	High-dose group	F/χ^2	P
First month	256.60±90.62	243.25±85.94	261.13±88.09	265.04±99.96	0.374	0.690
Third month	201.04±65.15	196.47±59.25	201.53±57.22	206.65±86.19	0.105	0.901
Sixth month	202.99±67.16	209.76±61.53	198.35±69.04	199.83±75.03	0.183	0.833
**Subgroup**						
**Age(year)**						
21-27 First month	263.95±90.58	256.20±98.74	281.67±71.33	257.89±99.83	0.283	0.755
Third month	213.66±54.84	218.04±62.48	221.74±51.81	190.82±47.47	0.658	0.527
Sixth month	210.56±62.71	219.72±53.27	202.98±50.31	206.24±86.38	0.224	0.801
28-35 First month	247.65±91.29	218.96±51.78	244.99±98.86	274.33±104.74	0.818	0.451
Third month	190.00±72.03	172.74±47.65	184.44±57.85	217.21±106.20	0.966	0.393
Sixth month	194.37±72.03	196.81±71.71	194.49±83.63	190.67±60.45	0.014	0.986
**Gestation (week)**						
37-39 First month	248.52±90.51	251.82±97.15	237.75±77.03	256.30±101.17	0.162	0.851
Third month	193.00±63.02	207.33±61.66	182.39±54.03	188.66±75.58	0.576	0.567
Sixth month	203.19±68.87	219.50±59.25	189.39±75.15	199.76±72.98	0.711	0.497
40-42 First month	270.59±90.85	223.66±53.34	296.20±95.83	278.62±102.51	1.412	0.264
Third month	217.13±67.97	174.74±51.38	228.34±52.73	278.63±104.28	3.419	0.057
Sixth month	202.49±64.72	191.49±65.53	214.04±58.06	200.30±124.59	0.222	0.804

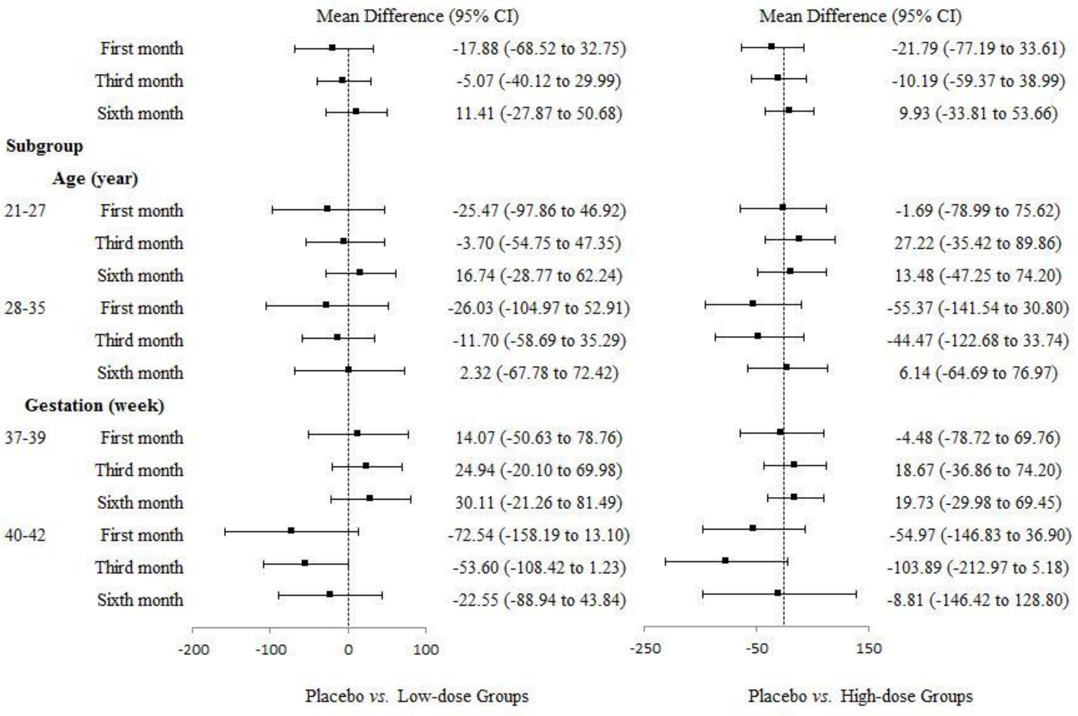

Figure S2. Mean difference of pigmentation between MSCs and placebo group.

Table S5. Erythema outcome among participants.

	All participants	Placebo group	Low-dose group	High-dose group	F/χ^2	P
First month	356.53±73.98	366.68±54.46	346.02±62.16	357.79±100.00	0.465	0.630
Third month	324.38±48.93	312.87±48.57	338.86±51.75	317.31±41.36	1.839	0.168
Sixth month	313.30±73.87	329.98±81.58	321.59±61.81	280.00±70.45	2.579	0.084
**Subgroup**						
**Age(year)**						
21-27 First month	356.60±73.98	364.27±53.99	351.59±72.46	351.99±97.15	0.125	0.883
Third month	335.29±47.97	321.23±48.88	353.09±48.95	328.45±40.82	1.322	0.285
Sixth month	325.24±81.16	353.77±80.45	327.87±61.88	285.52±89.59	2.150	0.134
28-35 First month	356.44±75.16	371.21±58.77	341.65±55.20	365.34±108.39	0.479	0.624
Third month	314.83±48.49	303.68±49.07	326.82±52.85	309.89±42.39	0.695	0.507
Sixth month	299.71±63.24	299.05±75.89	316.36±63.99	272.11±32.75	1.090	0.351
**Gestation (week)**						
37-39 First month	355.92±68.31	376.51±53.35	350.49±72.20	338.19±77.49	1.260	0.294
Third month	323.10±48.50	322.44±43.38	336.72±58.25	307.98±40.24	1.146	0.329
Sixth month	306.30±65.41	315.22±55.29	320.91±73.86	283.75±64.63	1.406	0.257
40-42 First month	357.59±84.32	344.23±53.97	339.32±45.95	388.28±126.59	0.912	0.416
Third month	326.94±50.96	293.74±56.07	341.86±43.87	354.67±21.22	2.807	0.088
Sixth month	330.40±91.21	357.65±116.02	322.79±36.46	251.85±138.38	1.146	0.344

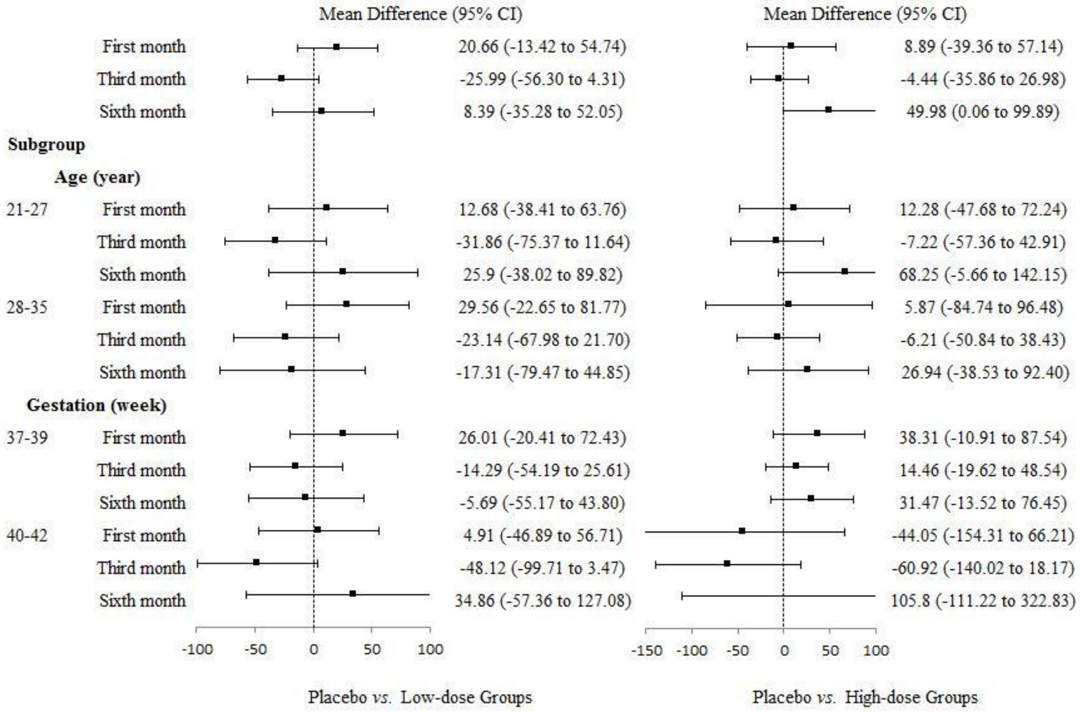

Figure S3. Mean difference of Erythema between MSCs and placebo group.

Table S6. Scar area (mm^2) outcome among participants.

	All participants	Placebo group	Low-dose group	High-dose group	F/χ^2	*P*
Scar Area (mm^2)						
First month	292.70±87.17	277.42±75.68	306.69±79.02	291.49±105.60	0.669	0.516
Third month	319.65±60.02	317.40±58.13	312.34±57.61	334.33±67.52	0.632	0.535
Sixth month	378.01±113.20	385.49±110.97	386.27±119.37	357.20±112.19	0.388	0.680
**Subgroup**						
**Age(year)**						
21-27 First month	299.33±100.02	288.22±78.69	326.21±108.93	289.30±114.34	0.541	0.587
Third month	315.69±63.07	332.59±51.96	313.04±73.25	292.38±62.06	0.764	0.477
Sixth month	365.39±135.08	414.49±134.98	327.55±132.45	339.41±131.22	1.479	0.244
28-35 First month	284.62±69.09	258.52±71.00	292.37±46.41	294.88±96.95	0.753	0.480
Third month	322.99±58.13	302.22±62.61	311.75±43.45	362.29±58.06	3.391	0.047
Sixth month	392.37±81.63	347.80±54.88	435.21±84.29	382.61±80.36	3.842	0.035
**Gestation (week)**						
37-39 First month	297.61±84.30	283.45±64.93	298.26±64.87	313.06±119.90	0.450	0.641
Third month	316.67±59.41	325.30±58.94	301.14±58.87	325.46±61.88	0.736	0.486
Sixth month	369.35±105.20	375.22±103.54	377.53±104.29	355.86±113.47	0.182	0.834
40-42 First month	283.65±93.32	261.35±104.80	320.18±100.00	257.93±72.27	1.313	0.289
Third month	325.44±62.33	302.74±58.03	328.03±54.82	369.80±92.18	1.267	0.307
Sixth month	399.17±131.60	404.75±128.87	401.58±148.77	367.23±144.63	0.060	0.942

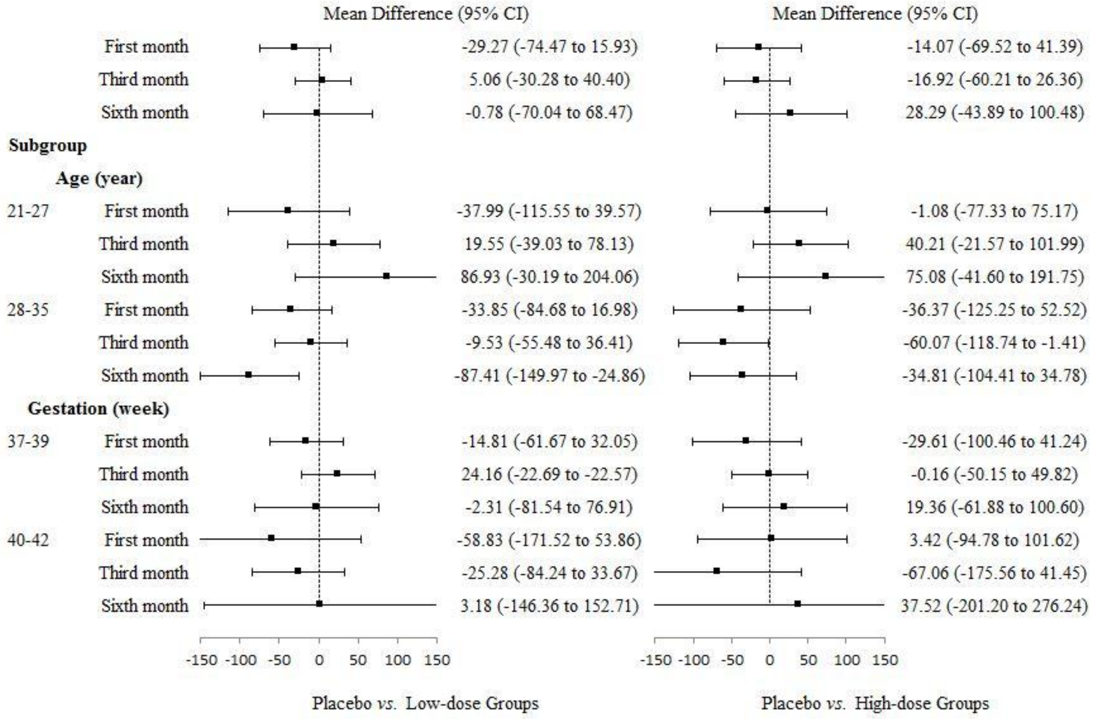

Figure S4. Mean difference of Scar Area (mm^2) between MSCs and placebo group.

Table S7. Scar volume (mm^3) outcome among participants.

	All participants	Placebo group	Low-dose group	High-dose group	F/χ^2	P
First month	694.18±316.69	663.26±286.55	742.97±302.38	668.59±363.85	0.481	0.620
Third month	699.91±233.29	712.92±223.20	667.50±235.50	734.40±251.83	0.418	0.660
Sixth month	837.84±407.10	876.02±390.03	895.92±441.52	711.02±378.14	1.156	0.322
**Subgroup**						
**Age(year)**						
21-27 First month	742.51±374.98	710.62±334.04	853.55±421.97	687.15±384.23	0.674	0.516
Third month	690.98±241.12	784.93±198.67	632.30±268.45	641.96±242.95	1.230	0.310
Sixth month	798.30±417.17	976.13±452.33	676.57±419.14	688.83±307.94	2.083	0.142
28-35 First month	635.27±218.16	280.38±163.63	661.88±139.40	639.72±350.17	0.351	0.707
Third month	707.44±230.07	640.91±232.76	697.29±210.11	796.03±251.81	1.105	0.345
Sixth month	882.84±397.77	745.88±256.44	1078.70±385.00	742.71±486.91	2.801	0.079
**Gestation (week)**						
37-39 First month	714.42±311.94	672.89±246.91	714.26±264.40	762.07±425.73	0.296	0.746
Third month	708.75±238.30	739.93±247.20	639.37±218.78	755.91±251.46	0.937	0.401
Sixth month	819.04±384.89	888.73±368.60	858.36±401.69	712.66±387.85	0.887	0.420
40-42 First month	656.92±328.41	637.58±401.09	788.91±365.58	523.16±174.54	1.649	0.215
Third month	682.66±228.23	662.78±176.16	706.89±263.90	648.30±286.81	0.106	0.900
Sixth month	883.79±465.65	852.20±453.20	961.64±526.69	698.74±426.10	0.263	0.772

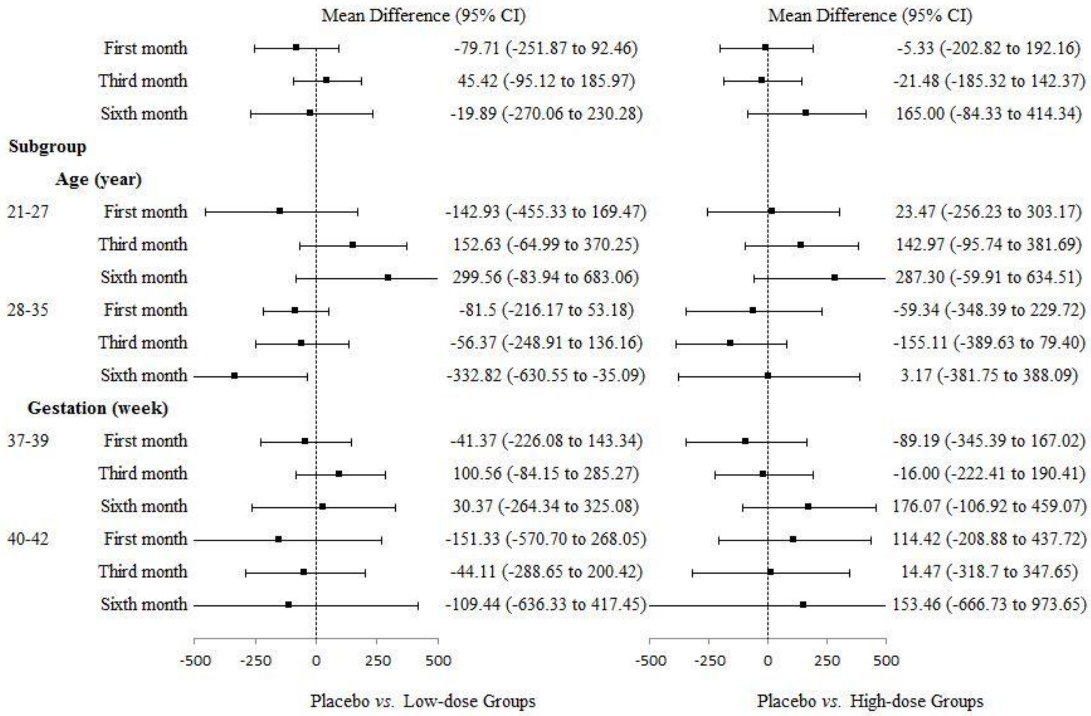

Figure S5. Mean difference of Scar volume (mm^3) between MSCs and placebo group.

Table S8. Immunoglobulins (IgG, IgA, IgM) and complements (C3, C4) in milk outcomes among participants.

	All participants	Placebo group	Low-dose group	High-dose group	F/χ^2	*P*
**Third day**						
IgG	0.42±0.18	0.39±0.17	0.41±0.12	0.44±0.22	0.535	0.588
IgA	3.73±1.20	3.47±1.54	3.92±0.87	3.82±1.09	1.091	0.341
IgM	0.94±0.25	0.93±0.37	0.93±0.14	0.96±0.19	0.067	0.936
C3	0.68±0.25	0.67±0.29	0.65±0.21	0.70±0.26	0.208	0.813
C4	0.54±0.21	0.56±0.31	0.55±0.12	0.52±0.13	0.230	0.795
**First month**						
IgG	0.54±0.80	0.37±0.07	0.77±1.31	0.44±0.14	1.554	0.220
IgA	3.72±1.09	3.65±0.78	3.52±1.02	3.99±1.35	1.137	0.328
IgM	0.97±0.20	0.97±0.19	0.93±0.17	1.02±0.24	1.055	0.354
C3	0.64±0.16	0.64±0.11	0.58±0.11	0.69±0.22	2.796	0.069
C4	0.54±0.13	0.53±0.09	0.52±0.06	0.57±0.19	1.058	0.354
**Third month**						
IgG	0.71±0.94	1.17±1.39	0.48±0.47	0.51±0.60	2.913	0.065
IgA	3.34±1.20	2.93±1.48	3.53±0.76	3.55±1.36	1.323	0.277
IgM	0.98±0.17	1.02±0.10	0.97±0.14	0.97±0.26	0.315	0.732
C3	0.58±0.17	0.56±0.10	0.54±0.07	0.67±0.29	2.362	0.106
C4	0.49±0.10	0.50±0.09	0.46±0.06	0.54±0.14	2.860	0.068
**Sixth month**						
IgG	0.49±0.75	0.32±0.06	0.31±0.09	2.04±2.28	8.48	0.003
IgA	3.10±0.93	3.37±0.90	3.03±0.65	2.20±2.00	1.429	0.267
IgM	0.88±0.17	0.89±0.13	0.90±0.14	0.82±0.47	0.177	0.840
C3	0.56±0.18	0.53±0.16	0.54±0.12	1.04±	6.428	0.009
C4	2.87±10.45	5.51±15.18	0.47±0.05	0.66±	0.519	0.605

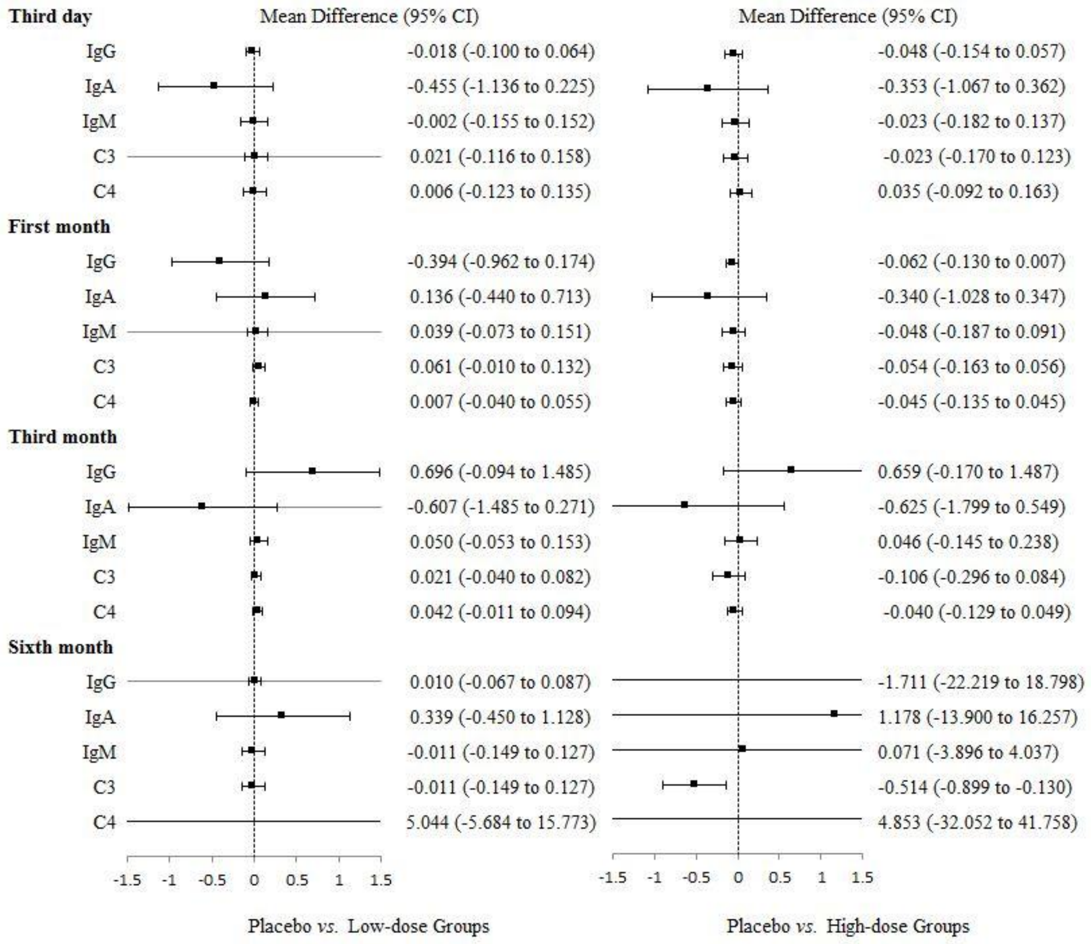

Figure S6. Mean difference of immunoglobulins (IgG, IgA, IgM) and complements (C3, C4) in milk between MSCs and placebo group.
